# Phase II study of gemcitabine plus oxaliplatin as first-line chemotherapy for advanced non-small-cell lung cancer

**DOI:** 10.1038/sj.bjc.6602667

**Published:** 2005-06-14

**Authors:** F Cappuzzo, S Novello, F De Marinis, V Franciosi, M Maur, A Ceribelli, V Lorusso, F Barbieri, L Castaldini, E Crucitta, L Marini, S Bartolini, G V Scagliotti, L Crinò

**Affiliations:** 1 Division of Medical Oncology, Bellaria Hospital, Bologna, Via Altura 3, 40139 Bologna, Italy; 2 Division of Pneumology, S Luigi Gonzaga Hospital, Regione Gonzole 10, 10043 Orbassano, Italy; 3 Pulmonary-Oncology Unit, Forlanini Hospital, Piazza Carlo Forlanini 1, 00151 Rome, Italy; 4 Division of Medical Oncology, Azienda Ospedaliera di Parma, Via Gramsci 14, 43100 Parma, Italy; 5 Division of Medical Oncology, Policlinico di Modena, Via Del Pozzo, 71, 41100 Modena, Italy; 6 Department of Medical Oncology A, Regina Elena Cancer Institute, Istituto Regina Elena 291, 00161 Rome, Italy; 7 Operative Unit of Medical Oncology, Oncology Institute of Bari, Via Samuele Hahnemann, 209 Bari, Italy; 8 Medical Division, Eli Lilly, Via Gramsci 731, 50019 Sesto Fiorentino, Florence, Italy

**Keywords:** oxaliplatin, gemcitabine, chemotherapy, non-small-cell lung cancer

## Abstract

This phase II study evaluated the response rate and tolerability of gemcitabine–oxaliplatin chemotherapy in non-small-cell lung cancer (NSCLC) patients. Chemonaive patients with stage IIIB or IV NSCLC received gemcitabine 1000 mg m^−2^ on days 1 and 8, followed by oxaliplatin 130 mg m^−2^ on day 1. Cycles were repeated every 21 days for up to six cycles. From February 2002 to May 2004, 60 patients were enrolled into the study in seven Italian institutions. We observed one complete response (1.7%) and 14 partial responses (23.3%), for an overall response rate of 25.0% (95% confidence interval, 14.7–37.9%). The median duration of response was 5.9 months (range 1.5–17.1 months). With a median follow-up of 6.7 months, median time to progressive disease and overall survival were 2.7 (range 1.9–3.4 months) and 7.3 months (range 7.2–8.6 months), respectively. The main grade 3–4 haematological toxicities were transient neutropenia in 11.7% and thrombocytopenia in 8.3% of the patients. Nausea/vomiting was the main grade 3–4 nonhaematological toxicity, occurring in 10.0% of the patients. Two (3.3%) patients developed grade 3 neurotoxicity. Our results show that gemcitabine–oxaliplatin chemotherapy is active and well tolerated in patients with advanced NSCLC, deserving further study, especially for patients not eligible to receive cisplatin.

Non-small-cell lung cancer (NSCLC) is the leading cause of cancer death in the world (Greenlee *et al*, 2001). Platinum-based chemotherapy is considered the standard treatment for advanced disease, with cisplatin considered the most effective for NSCLC (Non-Small Cell Lung Cancer Collaborative Group, 1995). New agents, such as taxanes and vinorelbine, administered with a platinum derivative, have resulted in improved median and 1-year survival times when compared with either cisplatin alone or older platinum-based combinations in randomised trials (Le Chevalier *et al*, 1994; Wozniak *et al*, 1998; Bonomi *et al*, 2000; Sandler *et al*, 2000).

Gemcitabine's mild toxicity profile, both in terms of haematological toxicity and visceral side effects, and novel mechanism of action have encouraged its use in combination with traditional agents, such as cisplatin. Gemcitabine–cisplatin chemotherapy is considered one of the most active regimens for advanced NSCLC, with an overall response rate (ORR) of 22–38% and median survival of 8.1–9.8 months in phase III trials (Crinò *et al*, 1999; Scagliotti *et al*, 2002; Schiller *et al*, 2002). Although several phase III trials of new platinum-based doublets (Kelly *et al*, 2001; Schiller *et al*, 2002; Smit *et al*, 2003) have produced similar results, a recent meta-analysis showed an absolute 1-year survival benefit of 3.9% for gemcitabine–cisplatin chemotherapy when compared to other platinum-containing regimens (Le Chevalier *et al*, 2005).

Cisplatin has been the most widely used platinum derivative, but it is known to yield significant toxicities, including severe nausea and vomiting, and renal toxicity, requiring adequate hydration, ototoxicity and neuropathy. Oxaliplatin is a diaminocyclohexane platinum compound, with a mechanism of action similar to that of cisplatin (Woynarowski *et al*, 2000), that has shown activity against a variety of cancer types, including colon (de Gramont *et al*, 1997) and NSCLC (Monnet *et al*, 1998). Oxaliplatin has a more manageable toxicity profile than cisplatin, with no renal toxicity and a lower incidence of haematological and gastrointestinal toxicities. It lacks the nephrotoxicity of cisplatin and causes less myelotoxicity than carboplatin, the leading cisplatin analog, and it may be active in cisplatin- and carboplatin-resistant tumours (Chollet *et al*, 1996; Raymond *et al*, 1998b). The main toxicity associated with oxaliplatin is a generally reversible peripheral neuropathy (Raymond *et al*, 1998a).

Oxaliplatin has demonstrated synergy with gemcitabine, with a sequence dependency that favours the administration of gemcitabine, followed by oxaliplatin (Faivre *et al*, 1999). A phase I trial, conducted by Shibata *et al* (2001), investigated the maximum tolerated dose of gemcitabine–oxaliplatin in patients with solid tumours. This study showed that oxaliplatin 130 mg m^−2^ on day 1 could be safely administered with gemcitabine 1000 mg m^−2^ on days 1 and 8 every 21 days. Using different schedules, other investigators (Faivre *et al*, 2002; Mavroudis *et al*, 2003) confirmed that gemcitabine–oxaliplatin is feasible, with no dose-limiting toxicities, easy to administer on an outpatient basis, and shows promising activity in patients with NSCLC and ovarian cancer.

On the basis of these data, we designed a phase II, multicentre trial to evaluate the response rate and tolerability of gemcitabine–oxaliplatin in untreated patients with stage IIIB or IV NSCLC, applying the same 3-week dosing schedule as per Shibata *et al*. Secondary end points were time to progressive disease (TtPD) and overall survival (OS).

## PATIENTS AND METHODS

### Patient selection

Chemonaive patients with histologically or cytologically confirmed locally advanced or metastatic stage IIIB or IV NSCLC not amenable to surgery or radiotherapy with curative intent were eligible for this study. Other eligibility criteria included the presence of at least one measurable lesion; age ⩾18 to ⩽75 years; Karnofsky performance status (KPS) ⩾70, life expectancy ⩾12 weeks; adequate bone marrow reserve (absolute neutrophil count ⩾2.0 × 10^9^ l^−1^, platelet count ⩾100 × 10^9^ l^−1^). Patients with asymptomatic brain metastases were eligible. No prior chemotherapy or immunotherapy or concomitant radiotherapy was allowed. Prior radiotherapy was acceptable if completed 4 weeks before study entry.

Exclusion criteria included inadequate liver function (bilirubin >1.5 times above normal range, aspartate transaminase (AST) and alanine transaminase (ALT) >3 times the normal range, or five times greater than the normal range in the presence of liver metastases); severe alteration of renal functionality (serum creatinine >2 × the upper limit of normal (ULN)); no second malignancies (except for carcinoma of the cervix uteri *in situ* and squamous cell carcinoma of the skin); peripheral neuropathy; untreated superior vena cava syndrome; hypercalcaemia requiring intravenous (i.v.) treatment; the presence of uncontrolled cardiac disease, uncontrolled diabetes mellitus, infections or other medical conditions that could have interfered with the trial.

Written informed consent was obtained from each patient before entering the study. The study was approved by the local Ethics Committee and was conducted in accordance with ethical principles stated in the most recent version of the Declaration of Helsinki or the applicable guidelines on good clinical practice, whichever represented the greater protection of the individual.

### Treatment plan and dose adjustments

Gemcitabine (Gemzar®, Eli Lilly and Company, Indianapolis, IN, USA) 1000 mg m^−2^ was administered as a 30-min i.v. infusion on days 1 and 8 of a 21-day cycle. Oxaliplatin (Eloxatin®, Sanofi-Synthelabo, Paris, France) 130 mg m^−2^ was administered as a 2-h i.v. infusion on day 1, following gemcitabine. All patients were scheduled to receive at least two cycles of therapy, and up to six cycles if there was no evidence of disease progression. Treatment was stopped early in cases of patient refusal, severe toxicity, documented progressive disease (PD) or pregnancy. Patients with PD after two or four cycles or with stable disease after four cycles of chemotherapy were withdrawn from the study. Full supportive therapy, corticosteroids, anticonvulsants and antibotics were given as needed. Antiemetic premedication included 5-HT_3_ antagonists. No routine use of haematopoietic growth factors was planned. No prophylactic antibiotics were used. All patients were treated on an outpatient basis.

Dose adjustments during treatment were based on haematological and nonhaematological toxicities. On day 1, if neutrophil count was <1.5 × 10^9^ l^−1^ and/or platelet count was <100 × 10^9^ l^−1^, chemotherapy doses were delayed (for up to 2 weeks) and doses were reduced by 25% to allow recovery from haematological toxicity. On day 8, for a neutrophil count <1.0 and/or platelets <75, the gemcitabine dose was omitted, and the cycle continued with one gemcitabine dose not given. Patients not recovering from haematological toxicity (neutrophil count >1.0 and platelets >75) within 2 weeks were withdrawn from the trial. Doses were reduced by 25% for any grade 3 nonhaematological toxicity (excluding nausea, vomiting and alopecia). Treatment was discontinued in the event of grade 4 or frequent grade 3 nonhaematological toxicity. For grade 2–4 neurological toxicity, oxaliplatin treatment was delayed until the patient recovered to grade 1, then the dose was reduced by 25%. If no recovery to grade 1 was achieved in 3 weeks, the patient was discontinued from the trial.

For hepatic toxicity, the day-1 gemcitabine dose was reduced by 25% for AST/ALT 1.6 to 5.0 × ULN, alkaline phosphatase ⩽5.0 × ULN and bilirubin <ULN. Treatment was delayed for up to 2 weeks for AST/ALT >5 × ULN or alkaline phosphatase >5 × ULN or bilirubin >ULN. The day-1 gemcitabine dose was omitted for a total bilirubin >1.5 ULN. For nephrotoxicity, gemcitabine doses were reduced by 25% for serum creatinine >1.5–2.0 ULN and creatinine clearance ⩾50 ml min^−1^. Treatment was delayed for serum creatinine >1.5 UNL and creatinine clearance <50 ml min^−1^ or serum creatinine greater than >2.0 UNL with any creatinine clearance value.

### Baseline and treatment assessments

Pretreatment evaluation included physical examination; KPS; chest X-ray; brain, thoracic and abdominal computer tomography scan (CT scan); bronchoscopy (if not performed at the time of diagnosis); bone scan; electrocardiogram; complete blood count and blood chemistry with liver function tests and creatinine clearance. On days 1 and 8, a physical exam (including weight) was performed, and KPS and blood count were assessed. All measurable and evaluable lesions were assessed by the same method used at baseline. Response to therapy was assessed every two cycles with clinical and/or radiological tumour assessment.

In responding patients, a confirmatory assessment was repeated after at least 4 weeks, according to the RECIST criteria (Therasse *et al*, 2000). Assessment of TtPD was determined by measuring the time interval from the beginning of treatment until the first documentation of progression or death due to any cause. Survival (OS) was determined by measuring the time interval from the beginning of the treatment to the date of death or last contact. Toxicity was evaluated according to NCI criteria (Common Toxicity Criteria, 1993). No quality of life questionnaire was used in the present study.

### Statistical plan

A two-stage design was used for the study. Using the Simon (1989) hypothesis, assuming a response rate of 40%, a probability of error of 5% and a power of 90%, a total of 54 patients were to be enrolled, with at least five responses to be noted in the first 19 patients. Treatment was considered effective if at least 16 objective responses were observed. Every patient included in the study was considered evaluable (intent-to-treat analysis). Response rates, including 95% confidence intervals (CIs), were calculated on an intent-to-treat basis. Time-to-event end points were calculated using the Kaplan–Meier method, with the appropriate censoring (Kaplan and Meier, 1958).

## RESULTS

### Patient characteristics

From February 2002 to May 2004, a total of 60 patients were enrolled in this trial in seven Italian centres. Patients’ characteristics at baseline are listed in Table 1. The majority of patients were male (71.7%), with a median age of 61 years (range 36–74). Performance status was 100 in 46.7% of patients; 51 (85.0%) patients had stage IV and nine (15.0%) had stage IIIB disease. Histology was predominantly adenocarcinoma (55.9% of the patients).

### Efficacy

**Figure 1:**
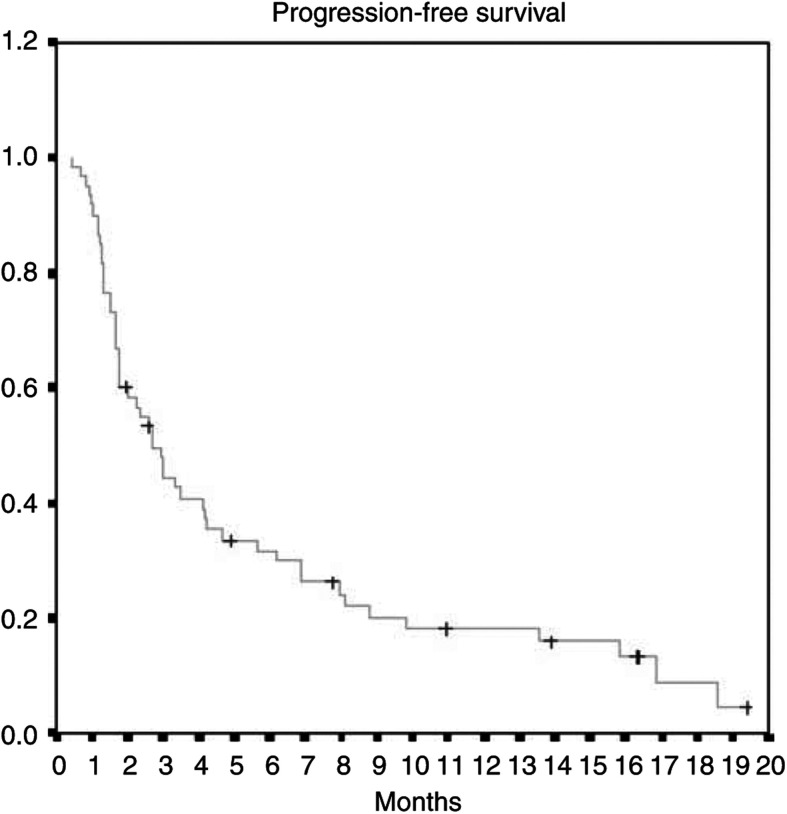
Kaplan–Meier curve for progression-free survival.

**Figure 2:**
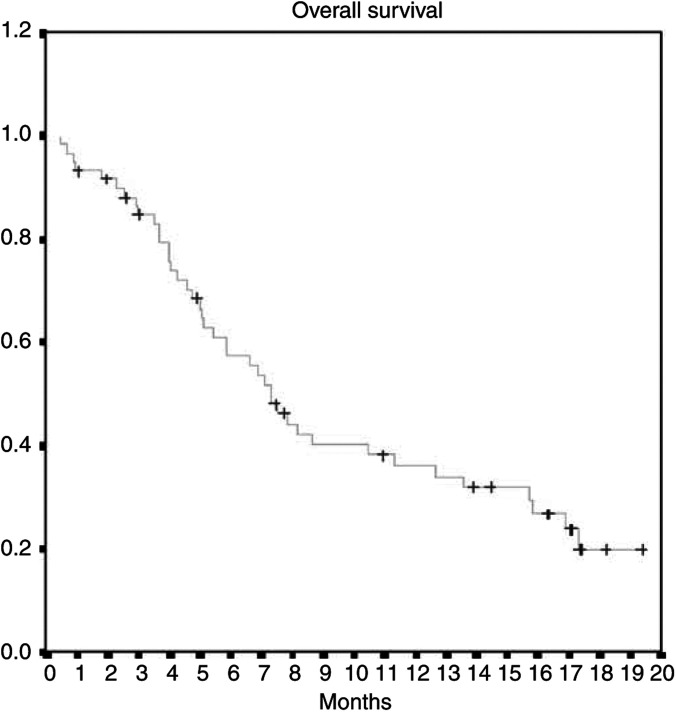
Kaplan–Meier curve for OS.

As per the ITT analysis, the ORR was 25.0% (95% CI, 14.7–37.9%), including one complete response (CR: 1.7%) and 14 partial responses (PRs: 23.3%, Table 2). Two patients were not assessable for response because they discontinued the trial after the first cycle, one for heart attack and the other for cardiac failure; neither event was related to the study drugs. The median duration of response was 5.9 months (range 1.5–17.1 months). With a median follow-up time of 6.7 months, the median TtPD was 2.7 (range 1.9–3.4 months), median OS time was 7.3 months (range 7.2–8.6 months) and 1-year survival was 36% (Figures 1, 2).

### Treatment administration

A total of 209 chemotherapy courses were administered, with a median of 3.9 cycles per patient (range 1–6), and 16 (26.7%) patients received all six cycles. The planned dose intensity was 666.6 mg m^−2^ week^−1^ for gemcitabine and 43.3 mg m^−2^ week^−1^ for oxaliplatin. Dose intensity for all 60 patients was 615 mg m^−2^ week^−1^ for gemcitabine and 42.4 mg m^−2^ week^−1^ for oxaliplatin, with 92.3% of the planned gemcitabine dose and 97.9% of the planned oxaliplatin dose delivered.

### Toxicity

The main haematological toxicities (Table 3) were transient grade 3–4 neutropenia observed in seven (11.7%). patients, grade 3–4 thrombocytopenia not requiring platelet transfusion in five (8.3%) patients and grade 3 anaemia in one (1.7%) patient. No neutropenic fever or bleeding episodes were recorded. Nonhaematological toxicity was generally manageable (Table 4). Grade 3 nausea/vomiting occurred in five (8.3%) and grade 4 occurred in one (1.7%) patients. Two (3.3%) patients developed grade 3 neurotoxicity requiring oxaliplatin dose reduction. Two (3.3%) patients had grade 4 cardiovascular toxicity consisting of extrasystoles and atrial flutter. A total of 25.0% of the patients displayed grade 1–2 pulmonary symptoms, such as dyspnea, but this was due to disease progression. Grade 1 nephrotoxicity occurred in one (1.7%) patient. Two serious adverse events were reported during the study. One patient died after the first course due to heart attack and the other patient died due to cardiac failure. Neither of these deaths was considered treatment-related.

## DISCUSSION

Our study confirmed the promising activity and favourable toxicity profile of gemcitabine–oxaliplatin in patients with stage IIIB/IV NSCLC. We observed an ORR of 25.0%, with one CR and 14 PRs. The median duration of response was 5.9 months (range 1.5–17.1 months). Median TtPD was 2.7 months (range 1.9–3.4 months) and median OS was 7.3 months (range 7.2–8.6 months).

The 25% ORR observed in the present study seems to be not different from the 20–22% response rate obtained with gemcitabine when used as single agent (Abratt *et al*, 1994; Gatzemeier *et al*, 1996). However, although our study population was relatively young and with good performance status, a large percentage of stage IV patients was enrolled, including individuals with unfavourable prognostic factors, such as brain metastases.

Two trials have evaluated the activity and toxicity of gemcitabine–oxaliplatin in NSCLC patients (Faivre *et al*, 2002; Franciosi *et al*, 2003). Both trials included pretreated patients, and response rates ranged from 16 to 34%. The 16% response rate reported in the Franciosi trial was lower than that reported in our study (25.0%), probably because our study was conducted only in chemonaive patients. In the study conducted by Faivre *et al*., 12 of the 35 patients (34.2%) enrolled in the trial responded to the therapy, including two platinum-resistant patients. These results are better than those observed in our trial, probably because of the different characteristics of our cohort, which included a larger percentage of stage IV disease (85% in our study *vs* 71%), and a lower percentage of patients with a performance status of 100 (46.7% in our study *vs* 85.7%).

The response rate observed with gemcitabine–oxaliplatin in the present trial was also comparable to the activity of oxaliplatin-based combinations observed in other phase II studies. Oxaliplatin has been evaluated in combination with vinorelbine (Monnet *et al*, 2001) and in combination with taxanes (Winegarden *et al*, 2004), producing response rates of 19 and 37%, respectively.

One of the most important findings of our trial is the low toxicity profile demonstrated by gemcitabine–oxaliplatin. In phase II trials, single-agent gemcitabine was administered at the dose of 1000–1250 mg m^−2^ on days 1, 8 and 15 (Anderson *et al*, 1994; Gatzemeier *et al*, 1996; Le Chevalier *et al*, 1997), or at the dose of 1250 mg m^−2^ on days 1 and 8 when used in combination with cisplatin (Castellano *et al*, 1998). Planned dose intensity of gemcitabine in our study was lower than those reported by others (Anderson *et al*, 1994; Gatzemeier *et al*, 1996; Le Chevalier *et al*, 1997; Castellano *et al*, 1998), providing an additional explication for the excellent toxicity profile demonstrated by this regimen. We used the 130 mg m^−2^ dose per cycle for oxaliplatin and 2000 mg m^−2^ dose per cycle for gemcitabine evaluated in other reports (Franciosi *et al*, 2003; Mavroudis *et al*, 2003), and further demonstrated that the haematological and nonhaematological toxicities with this regimen are generally mild or moderate. The tolerability of the regimen is underscored by the fact that we were able to deliver full doses of the planned therapy to the majority of patients. The main grade 3–4 haematological toxicities were transient neutropenia and thrombocytopenia (11.7 and 8.3% of patients, respectively). Nausea/vomiting was the main grade 3–4 nonhaematological toxicity, occurring in six (10.0%) patients. Only two (3.3%) patients developed significant (grade 3) neurotoxicity.

The low incidence of side effects in our trial is of particular relevance to clinical practice because it suggests that gemcitabine–oxaliplatin could be used in patients ineligible for standard platinum-based therapy, such as the elderly or unfit. At the present time, single-agent therapy is the standard treatment for elderly or unfit patients (Gridelli *et al*, 2003), but there is clear evidence that two-drug combinations are better than single-agent therapy (Sederholm, 2002; Lilenbaum *et al*, 2005), and platinum-based chemotherapy should be considered for selected elderly patients (Bunn and Lilenbaum, 2003).

In conclusion, our findings further demonstrate that gemcitabine–oxaliplatin is active and well tolerated in patients with advanced NSCLC. The favourable toxicity profile of this regimen also justifies its use in patients in whom cisplatin may not be feasible, such as the elderly or unfit. Oxaliplatin hopefully will become an alternative to cisplatin whose toxicity profile is not acceptable for many patients with NSCLC. In the era of targeted therapies, this combination deserves to be studied further in association with new agents, such as the antivascular endothelial growth factor bevacizumab, or the monoclonal antibody antiepidermal growth factor receptor cetuximab.

## Figures and Tables

**Table 1:** Baseline patient characteristics (*N*=60)

**Characteristics**	**No. of patients**
Median age (range), years	61 (36–74)
Male/female (%)	43 (71.7)/17 (28.3)

*KPS* (%)	
100	28 (46.7)
90–80	31 (51.7)
70	1 (1.7)

Stage IIIB/IV (%)	9 (15.0)/51 (85.0)

*Sites of metastasis*	
Liver	6
Brain	4
Bone	4
Nodes	22
Others	17

*No. of metastatic sites*	
1	31
⩾2	20

*Histology* (%)	
Adenocarcinoma	33 (55.0)
Squamous	17 (28.3)
Bronchioloalveolar	1 (1.7)
Undifferentiated	5 (8.3)
Other NSCLC	3 (5.0)
Unknown	1 (1.7)

KPS=Karnofsky performance status; NSCLC=non-small-cell lung cancer.

**Table 2:** Best overall response (*N*=60)

**Response**	***n* (%)**
ORR	15 (25.0)
CR	1 (1.7)
PR	14 (23.3)
SD	18 (30.0)
PD	25 (41.7)
Not assessable	2 (3.3)

ORR=overall response rate; CR=complete response; PR=partial response; SD=stable disease; PD=progressive disease.

**Table 3:** Worst grade 3–4 NCI haematological toxicity

	**By patient (*N*=60)**		**By cycle (*N*=209)**	
**Toxicity**	**Grade 3 *n* (%)**	**Grade 4 *n* (%)**	**Grade 3 *n* (%)**	**Grade 4 *n* (%)**
Neutropenia	6 (10.0)	1 (1.7)	16 (7.7)	1 (0.5)
Thrombocytopenia	4 (6.7)	1 (1.7)	10 (4.8)	1 (0.5)
Anaemia	1 (1.7)	0	1 (0.5)	0
Leukopenia	1 (1.7)	0	1 (0.5)	0

**Table 4:** Worst grade 3–4 NCI nonhaematological toxicity

	**By patient (*N*=60)**		**By cycle (*N*=209)**	
**Toxicity**	**Grade 1–2 *n* (%)**	**Grade 3–4 *n* (%)**	**Grade 1–2 *n* (%)**	**Grade 3–4 *n* (%)**
Gastrointestinal	37 (61.7)	6 (10.0)	126 (60.3)	9 (4.3)
Neurological	27 (45.0)	2 (3.3)	52 (24.9)	2 (1.0)
Hepatic	17 (28.3)	2 (3.3)	73 (34.9)	2 (1.0)
Dermatological	11 (18.3)	1 (1.7)	12 (5.7)	1 (0.5)
Cardiovascular	7 (11.7)	2 (3.3)	7 (3.3)	2 (1.0)
Pulmonary	15 (25.0)	1 (1.7)	31 (14.8)	1 (0.5)

## References

[bib1] Abratt RP, Bezwoda WR, Falkson G, Goedhals L, Hacking D, Rugg TA (1994) Efficacy and safety profile of gemcitabine in non-small-cell lung cancer: a phase II study. J Clin Oncol 12: 1535–1540 8040664 10.1200/JCO.1994.12.8.1535

[bib2] Anderson H, Lund B, Bach F, Thatcher N, Walling J, Hansen HH (1994) Single-agent activity of weekly gemcitabine in advanced non-small-cell lung cancer: a phase II study. J Clin Oncol 12: 1821–1826 8083706 10.1200/JCO.1994.12.9.1821

[bib3] Bonomi P, Kim K, Fairclough D, Cella D, Kugler J, Rowinsky E, Jiroutek M, Johnson D (2000) Comparison of survival and quality of life in advanced non-small-cell lung cancer patients treated with two dose levels of paclitaxel combined with cisplatin *versus* etoposide with cisplatin: results of an Eastern Cooperative Oncology Group trial. J Clin Oncol 18: 623–631 10653877 10.1200/JCO.2000.18.3.623

[bib4] Bunn Jr PA, Lilenbaum R (2003) Chemotherapy for elderly patients with advanced non-small-cell lung cancer. J Natl Cancer Inst 95: 341–343 12618492 10.1093/jnci/95.5.341

[bib5] Castellano D, Lianes P, Paz-Ares L, Hidalgo M, Guerra JA, Gomez-Martin C, Gomez H, Calzas J, Cortes-Funes H (1998) A phase II study of a novel gemcitabine plus cisplatin regimen administered every three weeks for advanced non-small-cell lung cancer. Ann Oncol 9: 457–459 9636840 10.1023/a:1008276507236

[bib6] Chollet P, Bensmaine MA, Brienza S, Deloche C, Cure H, Caillet H, Cvitkovic E (1996) Single agent activity of oxaliplatin in heavily pretreated advanced epithelial ovarian cancer. Ann Oncol 7: 1065–1070 9037366 10.1093/oxfordjournals.annonc.a010500

[bib7] Common Toxicity Criteria (1993) National Institute of Health, Cancer Therapy Evaluation Program Division of Cancer Treatment: Bethesda

[bib8] Crinò L, Scagliotti GV, Ricci S, De Marinis F, Rinaldi M, Gridelli C, Ceribelli A, Bianco R, Marangolo M, Di Costanzo F, Sassi M, Barni S, Ravaioli A, Adamo V, Portalone L, Cruciani G, Masotti A, Ferrara G, Gozzelino F, Tonato M (1999) Gemcitabine and cisplatin *versus* mitomycin, ifosfamide, and cisplatin in advanced non-small-cell lung cancer: a randomized phase III study of the Italian Lung Cancer Project. J Clin Oncol 17: 3522–3530 10550150 10.1200/JCO.1999.17.11.3522

[bib9] de Gramont A, Vignoud J, Tournigand C, Louvet C, Andre T, Varette C, Raymond E, Moreau S, Le Bail N, Krulik M (1997) Oxaliplatin with high-dose leucovorin and 5-fluorouracil 48 h continuous infusion in pretreated metastatic colorectal cancer. Eur J Cancer 33: 214–219 9135491 10.1016/s0959-8049(96)00370-x

[bib10] Faivre S, Le Chevalier T, Monnerat C, Lokiec F, Novello S, Taieb J, Pautier P, Lhomme C, Ruffie P, Kayitalire L, Armand JP, Raymond E (2002) Phase I–II and pharmacokinetic study of gemcitabine combined with oxaliplatin in patients with advanced non-small-cell lung cancer and ovarian carcinoma. Ann Oncol 13: 1479–1489 12196375 10.1093/annonc/mdf219

[bib11] Faivre S, Raymond E, Woynarowski JM, Cvitkovic E (1999) Supraadditive effect of 2′,2′-difluorodeoxycytidine (gemcitabine) in combination with oxaliplatin in human cancer cell lines. Cancer Chemother Pharmacol 44: 117–123 10412945 10.1007/s002800050955

[bib12] Franciosi V, Barbieri R, Aitini E, Vasini G, Cacciani GC, Capra R, Camisa R, Cascinu S (2003) Gemcitabine and oxaliplatin: a safe and active regimen in poor prognosis advanced non-small cell lung cancer patients. Lung Cancer 41: 101–106 12826318 10.1016/s0169-5002(03)00150-8

[bib13] Gatzemeier U, Shepherd FA, Le Chevalier T, Weynants P, Cottier B, Groen HJ, Rosso R, Mattson K, Cortes-Funes H, Tonato M, Burkes RL, Gottfried M, Voi M (1996) Activity of gemcitabine in patients with non-small cell lung cancer: a multicentre, extended phase II study. Eur J Cancer 32A: 243–248 8664035 10.1016/0959-8049(95)00444-0

[bib14] Greenlee RT, Hill-Harmon MB, Murray T, Thun M (2001) Cancer statistics, 2001. CA Cancer J Clin 51: 15–36 11577478 10.3322/canjclin.51.1.15

[bib15] Gridelli C, Perrone F, Gallo C, Cigolari S, Rossi A, Piantedosi F, Barbera S, Ferrau F, Piazza E, Rosetti F, Clerici M, Bertetto O, Robbiati SF, Frontini L, Sacco C, Castiglione F, Favaretto A, Novello S, Migliorino MR, Gasparini G, Galetta D, Iaffaioli RV, Gebbia V, MILES Investigators (2003) Chemotherapy for elderly patients with advanced non-small-cell lung cancer: the Multicenter Italian Lung Cancer in the Elderly Study (MILES) phase III randomized trial. J Natl Cancer Inst 95: 362–372 12618501 10.1093/jnci/95.5.362

[bib16] Kaplan EL, Meier P (1958) Nonparametric estimation from incomplete observations. J Am Stat Assoc 53: 457–481

[bib17] Kelly K, Crowley J, Bunn Jr PA, Presant CA, Grevstad PK, Moinpour CM, Ramsey SD, Wozniak AJ, Weiss GR, Moore DF, Israel VK, Livingston RB, Gandara DR (2001) Randomized phase III trial of paclitaxel plus carboplatin *versus* vinorelbine plus cisplatin in the treatment of patients with advanced non-small-cell lung cancer: a Southwest Oncology Group trial. J Clin Oncol 19: 3210–3218 11432888 10.1200/JCO.2001.19.13.3210

[bib18] Le Chevalier T, Brisgand D, Douillard JY, Pujol JL, Alberola V, Monnier A, Riviere A, Lianes P, Chomy P, Cigolari S (1994) Randomized study of vinorelbine and cisplatin *versus* vindesine and cisplatin *versus* vinorelbine alone in advanced non-small-cell lung cancer: results of a European multicenter trial including 612 patients. J Clin Oncol 12: 360–367 8113844 10.1200/JCO.1994.12.2.360

[bib19] Le Chevalier T, Gottfried M, Gatzemeier U, Shepherd F, Weynants P, Cottier B, Groen HJ, Rosso R, Mattson K, Cortes-Funes H, Tonato M, Burkes RL, Voi M, Ponzio A (1997) A phase II multicenter study of gemcitabine in non small cell lung cancers. Bull Cancer 84: 282–288 9207875

[bib20] Le Chevalier T, Scagliotti G, Natale R, Danson S, Rosell R, Stahel R, Thomas P, Rudd RM, Vansteenkiste J, Thatcher N, Manegold C, Pujol JL, van Zandwijk N, Gridelli C, van Meerbeeck JP, Crino L, Brown A, Fitzgerald P, Aristides M, Schiller JH (2005) Efficacy of gemcitabine plus platinum chemotherapy compared with other platinum containing regimens in advanced non-small-cell lung cancer: a meta-analysis of survival outcomes. Lung Cancer 47: 69–80 15603856 10.1016/j.lungcan.2004.10.014

[bib21] Lilenbaum RC, Herndon II JE, List MA, Desch C, Watson DM, Miller AA, Graziano SL, Perry MC, Saville W, Chahinian P, Weeks JC, Holland JC, Green MR (2005) Single-agent *versus* combination chemotherapy in advanced non-small-cell lung cancer: the cancer and leukemia group B (study 9730). J Clin Oncol 23: 190–196 15625373 10.1200/JCO.2005.07.172

[bib22] Mavroudis D, Pappas P, Kouroussis C, Kakolyris S, Agelaki S, Kalbakis K, Androulakis N, Souglakos J, Vardakis N, Nikolaidou M, Samonis G, Marselos M, Georgoulias V (2003) A dose-escalation and pharmacokinetic study of gemcitabine and oxaliplatin in patients with advanced solid tumors. Ann Oncol 14: 304–312 12562660 10.1093/annonc/mdg063

[bib23] Monnet I, Brienza S, Hugret F, Voisin S, Gastiaburu J, Saltiel JC, Soulie P, Armand JP, Cvitkovic E, de Cremoux H (1998) Phase II study of oxaliplatin in poor-prognosis non-small-cell lung cancer (NSCLC). ATTIT. Association pour le Traitement des Tumeurs Intra Thoraciques. Eur J Cancer 34: 1124–1127 9849465 10.1016/s0959-8049(98)00007-0

[bib24] Monnet I, Soulie P, de Cremoux H, Saltiel-Voisin S, Bekradda M, Saltiel JC, Brain E, Dupont-Andre G, Cvitkovic E (2001) Phase I/II study of escalating doses of vinorelbine in combination with oxaliplatin in patients with advanced non-small-cell lung cancer. J Clin Oncol 19: 458–463 11208839 10.1200/JCO.2001.19.2.458

[bib25] Non-Small Cell Lung Cancer Collaborative Group (1995) Chemotherapy in non-small cell lung cancer: a meta-analysis using updated data on individual patients from 52 randomised clinical trials. Br Med J 311: 899–909 7580546 PMC2550915

[bib26] Raymond E, Chaney SG, Taamma A, Cvitkovic E (1998a) Oxaliplatin: a review of preclinical and clinical studies. Ann Oncol 9: 1053–1071 9834817 10.1023/a:1008213732429

[bib27] Raymond E, Faivre S, Woynarowski JM, Chaney SG (1998b) Oxaliplatin: mechanism of action and antineoplastic activity. Semin Oncol 25: 4–12 9609103

[bib28] Sandler AB, Nemunaitis J, Denham C, von Pawel J, Cormier Y, Gatzemeier U, Mattson K, Manegold C, Palmer MC, Gregor A, Nguyen B, Niyikiza C, Einhorn LH (2000) Phase III trial of gemcitabine plus cisplatin *versus* cisplatin alone in patients with locally advanced or metastatic non-small-cell lung cancer. J Clin Oncol 18: 122–130 10623702 10.1200/JCO.2000.18.1.122

[bib29] Scagliotti GV, De Marinis F, Rinaldi M, Crino L, Gridelli C, Ricci S, Matano E, Boni C, Marangolo M, Failla G, Altavilla G, Adamo V, Ceribelli A, Clerici M, Di Costanzo F, Frontini L, Tonato M, Italian Lung Cancer Project (2002) Phase III randomized trial comparing three platinum-based doublets in advanced non-small-cell lung cancer. J Clin Oncol 20: 4285–4291 12409326 10.1200/JCO.2002.02.068

[bib30] Schiller JH, Harrington D, Belani CP, Langer C, Sandler A, Krook J, Zhu J, Johnson DH, Eastern Cooperative Oncology Group (2002) Comparison of four chemotherapy regimens for advanced non-small-cell lung cancer. N Engl J Med 346: 92–98 11784875 10.1056/NEJMoa011954

[bib31] Sederholm C (2002) Gemcitabine *versus* gemcitabine/carboplatin in advanced non-small cell lung cancer: preliminary findings in a phase III trial of the Swedish Lung Cancer Study Group. Semin Oncol 29: 50–54 10.1053/sonc.2002.34276 12094340

[bib32] Shibata S, Chow W, Frankel P, Leong L, Lim D, Longmate J, Margolin K, McNamara M, Morgan R, Raschko J, Somlo G, Synold T, Tetef M, Twardowski P, Yen Y, Doroshow JH, Lenz H, Gandara D (2001) A phase I trial of oxaliplatin (OX) in combination with gemcitabine (G): A California Consortium Trial. Proc Am Soc Clin Oncol 20: A381

[bib33] Simon R (1989) Optimal two-stage designs for phase II clinical trials. Control Clin Trials 10: 1–10 2702835 10.1016/0197-2456(89)90015-9

[bib34] Smit EF, van Meerbeeck JP, Lianes P, Debruyne C, Legrand C, Schramel F, Smit H, Gaafar R, Biesma B, Manegold C, Neymark N, Giaccone G, European Organization for Research and Treatment of Cancer Lung Cancer Group (2003) Three-arm randomized study of two cisplatin-based regimens and paclitaxel plus gemcitabine in advanced non-small-cell lung cancer: a phase III trial of the European Organization for Research and Treatment of Cancer Lung Cancer Group – EORTC 08975. J Clin Oncol 21: 3909–3917 14581415 10.1200/JCO.2003.03.195

[bib35] Therasse P, Arbuck SG, Eisenhauer EA, Wanders J, Kaplan RS, Rubinstein L, Verweij J, Van Glabbeke M, van Oosterom AT, Christian MC, Gwyther SG (2000) New guidelines to evaluate the response to treatment in solid tumors. European Organization for Research and Treatment of Cancer, National Cancer Institute of the United States, National Cancer Institute of Canada. J Natl Cancer Inst 92: 205–216 10655437 10.1093/jnci/92.3.205

[bib36] Winegarden JD, Mauer AM, Otterson GA, Rudin CM, Villalona-Calero MA, Lanzotti VJ, Szeto L, Kasza K, Hoffman PC, Vokes EE, University of Chicago Phase II Network; Ohio State University (2004) A phase II study of oxaliplatin and paclitaxel in patients with advanced non-small-cell lung cancer. Ann Oncol 15: 915–920 15151948 10.1093/annonc/mdh215

[bib37] Woynarowski JM, Faivre S, Herzig MC, Arnett B, Chapman WG, Trevino AV, Raymond E, Chaney SG, Vaisman A, Varchenko M, Juniewicz PE (2000) Oxaliplatin-induced damage of cellular DNA. Mol Pharmacol 58: 920–927 11040038 10.1124/mol.58.5.920

[bib38] Wozniak AJ, Crowley JJ, Balcerzak SP, Weiss GR, Spiridonidis CH, Baker LH, Albain KS, Kelly K, Taylor SA, Gandara DR, Livingston RB (1998) Randomized trial comparing cisplatin with cisplatin plus vinorelbine in the treatment of advanced non-small-cell lung cancer: a Southwest Oncology Group study. J Clin Oncol 16: 2459–2465 9667264 10.1200/JCO.1998.16.7.2459

